# Attitudes and Knowledge of Medical Students Towards Healthcare for Lesbian, Gay, Bisexual, and Transgender Seniors: Impact of a Case-Based Discussion With Facilitators From the Community

**DOI:** 10.7759/cureus.17425

**Published:** 2021-08-25

**Authors:** Arkene Levy, Samiksha Prasad, Daniel P Griffin, Maria Ortega, Chasity B O’Malley

**Affiliations:** 1 Medical Education/Pharmacology, Nova Southeastern University Dr. Kiran C. Patel College of Allopathic Medicine, Fort Lauderdale, USA; 2 Medical Education/Microbiology, Nova Southeastern University Dr. Kiran C. Patel College of Allopathic Medicine, Fort Lauderdale, USA; 3 Medical Education, Nova Southeastern University Dr. Kiran C. Patel College of Allopathic Medicine, Fort Lauderdale, USA; 4 Medical Education/Physiology, Nova Southeastern University Dr. Kiran C. Patel College of Allopathic Medicine, Fort Lauderdale, USA

**Keywords:** sensitization, lgbt seniors, healthcare, undergraduate medical education, case-discussions, lgbt, diversity

## Abstract

Background: Lesbian, gay, bisexual, and transgender (LGBT) seniors are generally a medically underserved population that faces unique healthcare challenges. When compared to younger patients, LGBT seniors are at a greater risk for social isolation and have higher rates of smoking, disability, physical and mental distress, and lack of access to healthcare services. They are often reluctant to discuss their sexual orientations and gender identities with healthcare providers due to fear of discrimination and receiving inferior care based on prior unsatisfactory experiences with untrained or insensitive healthcare providers. Furthermore, recent research has revealed that only about 50% of primary care providers indicated confidence in providing culturally competent LGBT healthcare, highlighting the need for more LGBT proficiency training in medical school curricula.

Objectives: The aim of this study was to provide early intervention training to first-year medical students regarding best practices for equitable healthcare for LGBT seniors through integrative, small group, case-based discussions. The impact of this activity on the knowledge and attitudes of medical students regarding LGBT healthcare was also assessed.

Methods: First-year medical students participated in a two-hour small group, case-based discussion. Each group consisted of seven to eight students with one of seven facilitators who were invited members of the LGBT community. Students were provided with two clinical case scenarios related to treatment of LGBT senior patients. Students were given a pre/post-session knowledge and attitude survey to assess the impact of the session on their attitudes and understanding of the importance of providing equitable healthcare to LGBT patients. A rubric was also used by facilitators to evaluate level of student engagement and professionalism.

Results: A total of 51 first-year medical students attended the session and 38 (74.5%) completed the pre/post surveys. There was diverse representation in our student demographic with 5.2% of respondents identifying as LGBT. Survey results showed a significant increase in knowledge confidence and attitudes following the session. Students’ attitudes regarding determinants of health status changed significantly for nine of the 13 (69%) survey items. In addition, their confidence in knowledge regarding healthcare barriers, health issues, and practices for LGBT culturally competent care significantly increased post-session. Data from our assessment rubrics also show that students were highly professional and engaged with the LGBT facilitators.

Conclusion: Our study provides some evidence that case-based training of medical students regarding issues that affect health of LGBT seniors can improve attitudes and sensitize them to the unique needs of this population. Through this activity, the students indicated their desire to learn more about the topics covered and to receive further training in this field of study. While the study was somewhat limited by a small participant number, the significance of the data demonstrates the effectiveness of the approach involving members of the LGBT community as facilitators. Future work with these students as part of a longitudinal curriculum will include additional LGBT proficiency training to be offered in the subsequent blocks of instruction. Additionally, this intervention could potentially be adapted by other medical schools.

## Introduction

Approximately 5.6% of adults in the United States (US) identify as LGBT [1] and there are an estimated 2.7 million LGBT adults aged 50 or older living across the country [2]. LGBT seniors are generally an underserved and understudied population, who face unique challenges when seeking healthcare. When compared to younger patients, LGBT seniors are at a greater risk for social isolation and have higher rates of smoking, disability, physical and mental distress, and lack of access to healthcare services [3]. Many LGBT seniors report that their primary healthcare providers do not know about their sexual orientations, and many feel reluctant to discuss their sexual orientations and gender identities with healthcare providers due to fear of being judged or receiving inferior care [4,5]. Healthcare providers who fail to appropriately recognize sexual orientation not only reduce the confidence of patients in the healthcare system but also may overlook important preventative care procedures such as HIV screening [6]. Most importantly, more than 25% of older LGBT adults report concerns regarding discrimination as they age and only 50% expressed confidence that healthcare providers will treat them in a dignified manner [7].

There is a need therefore to enhance the cultural competencies of healthcare providers to reduce health inequalities between cisgender and LGBT people, and to enable more positive health outcomes for this population [8,9]. Healthcare providers who have not been trained in providing culturally competent care or sensitized to the health disparities faced by LGBT people are more likely to have difficulty with discussing sexuality, sexual orientation, and gender identity with their patients [10].

Recent research has revealed that only about 50% of participating primary care providers indicated confidence in providing culturally competent LGBT healthcare [6]. Data also shows that physicians are often wary of offending their patients or losing their trust and hence they avoid asking important questions surrounding gender identity and sexual orientation [11].

These issues and practices contribute to the health disparities and sub-optimal care experienced by senior LGBT individuals when compared to their cis-gendered counterparts [12,13]. One strategy to improve the cultural competency of future physicians is to provide targeted educational opportunities within medical school curricula for medical students to interact with the LGBT population through both simulated and actual scenarios [14]. These curricular interventions are effective at increasing the level of comfort, knowledge, and confidence that student doctors have for providing care to LGBT patients [14-16].

Therefore, the overarching goal of this study was to provide early integration of LGBT curriculum into the training of first-year medical students at the Nova Southeastern University Dr. Kiran C Patel College of Allopathic Medicine (NSU MD). We implemented this session in the winter semester of the first year of the curriculum. At this time, students are engaged in an organ systems course on the gastrointestinal system, human nutrition, endocrine and reproductive systems (GIHNER) and the second block of their pre-clerkship clinical skills longitudinal training in the course called Practice of Medicine 2 (PoM2). Both courses have been integrated to align topics. This curricular intervention included small group, case-based discussions facilitated by members of the LGBT community, using clinical scenarios that enabled discussion around best practices for providing equitable healthcare to LGBT seniors. We also assessed the impact of this activity on knowledge, confidence, and attitudes of medical students regarding LGBT healthcare using pre/post-session surveys.

## Materials and methods

The study was approved by the Nova Southeastern University Institutional Review Board (IRB #: 2020-298). Fifty-one first-year medical students at NSU MD participated in the session.

Session structure and context

The two-hour active learning session was embedded into the reflection, integration, and assessment (RIA) week at the end of the GIHNER course, and approximately the middle of the PoM2 course for first-year medical students at NSU MD, which takes place in the winter semester of year one of the curriculum. This was strategically placed as students had covered the reproductive and endocrine systems in problem and team-based learning and clinical skills sessions. Students also had opportunities to discuss bias and the LGBT patient during an interprofessional session with other health professions students at NSU. There were seven small groups with seven to eight students per group in zoom breakout rooms. Zoom breakout rooms were used instead of in-person small-group rooms due to safety concerns during the COVID-19 pandemic. Each group’s discussion was facilitated by a visiting member of the LGBT community. An NSU MD faculty member was also assigned to each group to assist with technical or professional issues, if necessary. The session, which included a 10-minute break scheduled by each group individually based on their progress in the session, was structured as follows: (i) 10 minutes for introductions of visiting community facilitators and pre-session survey, (ii) 40 minutes for discussing case 1, (iii) 40 minutes for discussing case 2, (iv) 20 minutes for a large group debrief and post-session survey, and (v) pre-designed 'prompt' questions by the LGBT community facilitators to stimulate engagement as students discussed the cases. These prompt questions were developed by the research team in collaboration with the LGBT community facilitators. The LGBT community facilitators also offered perspectives and shared lived experiences related to the scenarios as appropriate. 

Development of teaching materials

Two clinical case scenarios were designed to depict settings in which LGBT patients were treated either appropriately or inappropriately. The cases were developed with collaborative input from the LGBT facilitators over the course of three months prior to the session. Facilitators shared their lived experiences including physician interactions; religious affiliations; bias and discrimination experiences; and educational resources related to LGBT health and healthcare. This information was incorporated into the two cases to make them as representative of real experiences as possible. In brief, the first case presented the experience of a senior gay patient who identified as Latin-X and was a member of the Catholic community who was mistreated by his new primary care physician on his annual wellness visit. The second case presented the experience of a transgender female patient who was not called by her preferred pronoun by the nurse in the waiting room at her primary care physician’s office. 

Facilitators

The seven LGBT community facilitators identified as either lesbian, gay, and/or transgender. Facilitators were primarily recruited through the Sunshine Social Services organization (SunServe) in South Florida and personal networks and were offered compensation for their time. SunServe is a non-profit organization that provides critical life assistance and professional mental health services to LGBT youth, adults, and seniors in the greater South Florida metropolitan area. Before the session, the research team discussed with each facilitator the best strategies for sharing personal stories with the medical students. Facilitators also received a facilitator version of the cases prior to the session that guided group facilitation and prompt questions to stimulate discussion. Possible answers to prompt questions were also included in the facilitator guide.

Session assessment

After consenting to participate, students completed a 17-item attitude and knowledge confidence pre-survey before discussing the first case. The post-session survey was administered at the end of the discussion of both cases to assess changes in attitude, knowledge, and confidence regarding determinants of health status and healthcare challenges that face senior LGBT individuals. For each survey, students responded to statements using a five-point Likert scale from 'strongly disagree' to 'strongly agree'. The survey items were adopted with modifications from Gavzy et al. (2019) [17]. Survey instruments are appended as Appendix A. Survey data were paired and deidentified by a third party before analysis by the research team.

Facilitators also used a customized assessment rubric (Appendix B) tailored to qualitatively assess students’ ability to appropriately engage in discussion; their level of awareness and professionalism; and their comfort regarding discussing healthcare for the LGBT population. Rubrics were analyzed for professionalism issues by a third party and deidentified before sharing with the research team. 

Statistical Analysis

A paired students’ t-test was used to assess the statistical significance of the data obtained from the pre and post-session surveys, with a p-value less than 0.05 being considered as significant.

## Results

Participant demographic data

A total of 51 first-year medical students attended the session and of these, 38 (74.5%) students consented and completed the pre/post-session surveys. There was diverse representation in our student demographic (Table 1) with 31.58% identifying as male, 65.79% as female, and 2.63% as other. For sexual orientation, 92.11% identified as heterosexual, 5.2% identified as LGBT, and 2.63% identified as other. For the age ranges, 47.37% identified as 24 or younger, 42.11% as 25-29, 7.89% as 30-34, and 2.63% as 35-44. For ethnicity, 44.74% identified as white, non-Hispanic; 5.26% as black, non-Hispanic; 15.79% as Hispanic; 28.95% as Asian/Pacific Islander; and 5.26% as other.

 

**Table 1:** Demographic characteristics of the total participants (n=38) who completed the pre/post-session surveys. Data is represented as a percentage of the total participants.

Demographic Information of Participants							
Gender (%)		Sexual Orientation (%)		Age Range (Years)		Ethnicity (%)	
Female	65.79	Heterosexual	92.11	< 24	47.37	White, Non-Hispanic	44.74
Male	31.58	Gay	2.63	25-29	42.11	Hispanic	15.79
Other	2.63	Queer	2.63	30-34	7.89	Asian/Pacific Islander	28.95
		Other	2.63	35-44	2.63	Black, Non-Hispanic	5.26
						Other	5.26

Pre/Post-session survey analysis

**Figure 1:**
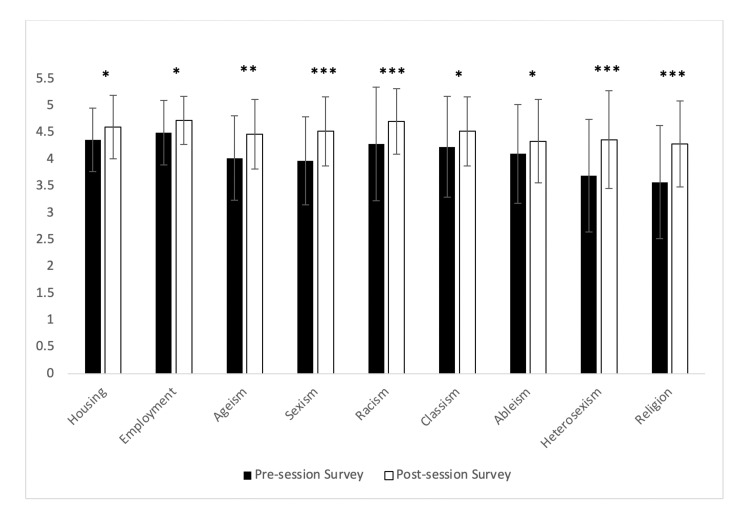
Change in student attitude Range of significance of results of the pre/post survey responses (n=38) to the following prompt: 'Please indicate the extent to which you agree or disagree that each of the following factors is a strong determinant of the health status of an individual or population.' Students responded on a 5-point Likert scale with 5 = strongly agree, 4 = agree, 3= undecided, 2 = disagree, 1 = strongly disagree. Data are represented as the average response from the Likert scale +/- the standard deviation. * represents p-value of < 0.05 ** represents p-value < 0.01 *** represents p-value < 0.001

For the pre/post-session surveys, the students responded to the following prompt on a Likert scale of 5 indicating 'strongly agrees', 4 'agrees', 3 'undecided', 2 'disagrees', and 1 'strongly disagrees': *Please indicate the extent to which you agree or disagree that each of the following factors is a strong determinant of the health status of an individual or population.* Students showed a positive increase in or no change to their level of agreement to all the determinants. A significant increase in agreement occurred in the following areas after the session: housing, employment, ageism, sexism, racism, classism, ableism, heterosexism, and religion (Table 2; Figure 1). Other aspects which were not significant included: lifestyle, access to healthcare, and educational status (Table 2).

**Table 2:** Pre- and post-session survey results of student agreement/disagreement with possible determinants of health status. A 5-point Likert scale was used with 5 indicating 'Strongly Agree' and 1 indicating 'Strongly Disagree'.  There was a significant positive change in agreement for nine of the thirteen options.  There was either no change or stronger agreement for all thirteen options. Housing indicates independent/assisted living SD: Standard deviation.

Social Determinants of Health Status	Pre-Survey	SD	Post-Survey	SD	p-value
Lifestyle	4.66	0.48	4.68	0.47	>0.1
Access to health care	4.89	0.31	4.89	0.31	1
Housing	4.27	0.59	4.61	0.59	<0.05
Employment	4.50	0.60	4.73	0.45	<0.05
Educational status	4.42	0.55	4.53	0.60	0.10
Ageism	4.03	0.79	4.47	0.65	<0.01
Sexism	3.97	0.82	4.53	0.64	<0.001
Racism	4.29	1.06	4.71	0.61	<0.001
Classism	4.24	0.94	4.53	0.65	<0.05
Ableism	4.10	0.92	4.34	0.78	<0.05
Heterosexism	3.70	1.05	4.37	0.91	<0.001
Religion	3.58	1.06	4.29	0.80	<0.001

**Figure 2:**
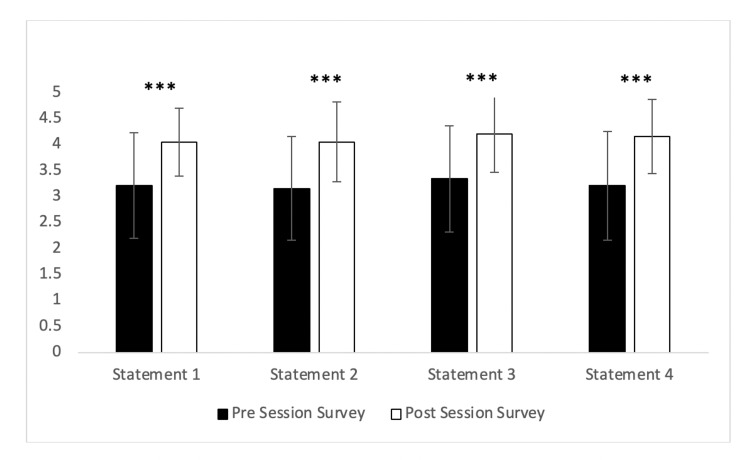
Change in student confidence Results of the pre/post survey responses  (n=38) to the following statements:​ Statement 1: I am confident in my knowledge about the systemic barriers to health faced by LGBT individuals.​; Statement 2: I am confident in my knowledge about the unique health issues and disparities for LGBT individuals.​; Statement 3: I am confident in my knowledge about good practices for promoting culturally competent care for LGBT individuals.​; Statement 4: I am confident in my knowledge about common inappropriate practices that prevent culturally competent care for LGBT individuals.​ Students responded on the following Likert scale: ​ 5 = strongly agree, 4 = agree, 3= undecided, 2 = disagree, 1 = strongly disagree. ​Data is represented as the average response from a Likert scale +/- the standard deviation. *** represents p-value < 0.001.

In response to the following statements, students showed a significant positive increase in all four areas of confidence examined (Figure 2). Statement 1: I am confident in my knowledge about the systemic barriers to health faced by LGBTQ+ individuals​ (3.21 +/- 1.02 pre- vs 4.05 +/- 0.66 post-, p< 0.001); Statement 2: I am confident in my knowledge about the unique health issues and disparities for LGBTQ+ individuals​ (3.16 +/- 1.00 pre- vs 4.05 +/- 0.77 post-, p< 0.001); Statement 3: I am confident in my knowledge about good practices for promoting culturally competent care for LGBT individuals​ (3.34 +/- 1.02 pre- vs 4.21 +/- 0.74 post-, p< 0.001); Statement 4: I am confident in my knowledge about common inappropriate practices that prevent culturally competent care for LGBT individuals (3.21 +/- 1.04 pre- vs 4.16 +/- 0.72 post-, p< 0.001). In the free narrative response section, seven students added information and 100% of those respondents requested further information regarding more training and information.

LGBT community facilitator evaluation of student performance

LGBT community facilitators were provided a customized narrative rubric for evaluating student performance during the session. Qualitative analysis of the rubrics indicated that the students maintained professionalism, demonstrated appropriate interpersonal and communicational skills, and made quality contributions to the discussion and prompt questions. Facilitators commented that students exhibited a high level of engagement with both faculty and visiting LGBT facilitators during the case discussions.

## Discussion

Our results showed significant improvements in knowledge confidence and attitudes regarding LGBT health and healthcare issues following the session. This is remarkable given that the data was found to be highly significant with a relatively small participant number. Based on feedback from both our faculty and LGBT facilitators, we believe that the involvement of members of the LGBT community contributed to the exceptional effectiveness of the session.

Our baseline data showed that students already had positive attitudes towards senior LGBT individuals. The senior LGBT community has most likely faced a lifetime of discrimination based on their gender identity and sexual orientation, so it is important to recognize that our future physicians at an early point in their training show positive attitudes that can be further developed and nurtured during their training to better serve the community health needs. We also learned during the large group debrief and from the feedback given by the facilitators, that the design of the clinical scenarios further exposed students to new types of interactions and stimulated their interest to have deeper discussions about both sexual orientation and gender identity, especially with members of the transgender community. 

Comparable studies with similar curricular interventions have reported that medical students when paired with members of the LGBT community for case-based or other active learning sessions are more engaged and comfortable with discussing issues related to the sexual orientation and gender identity of these individuals [18,19]. One good example is from the Columbia University Vagelos College of Physicians and Surgeons that implemented a two-hour curriculum for medical students, which included a panel with LGBT identified individuals [19]. The post-curriculum survey results from this intervention showed more positive attitudes among participants as well as an increase in correct answers for medical knowledge questions. Our curricular intervention was unique in that our clinical scenarios focused specifically on the disparities faced by LGBT seniors, and there is a paucity of data in the current literature describing such focused interventions. This focused area of training is to be emphasized as another component of the well-needed advanced LGBT curriculum for medical students in both allopathic and osteopathic medical schools across the US and Canada [20,21].

Overall, there are several implications of, and lessons learned from, our study. We identified an opportunity in our newly established curriculum to integrate training regarding senior LGBT healthcare knowledge and attitudes. We developed an intervention that not only increased students’ knowledge of LGBT healthcare and the challenges faced but also improved their attitude toward working with senior LGBT patients and confidence to care for this population. However, we recognize that more resources and training are needed to help our medical students recognize the unique healthcare challenges faced by LGBT seniors. Our single case-based session was an effective first step based on current practices in well-established medical schools [22]. Subsequent curricular content will include lectures and workshops led by authorities in the field of geriatric LGBT healthcare and clinical skills activities integrated into our practice of medicine sessions through the engagement of senior LGBT individuals as standardized patients. 

One limitation of this study is that students did not receive any formal LGBT terminology training prior to the activity. For future revisions of this activity, we plan to provide prior LGBT proficiency training so that students can apply that knowledge when discussing the cases. Another limitation is the small number of participants reduces the generalizability of the study to other medical programs. However, this was a pilot study and the intention is to expand the number of participants in subsequent sessions. Approximately 75% of the class volunteered to participate in the surveys. If all 51 students had participated in the surveys, it could have provided more insights into the session's effectiveness. One strategy to address the level of participation in the future will be to host a brief pre-session discussion the week before the activity. This will offer students an opportunity to learn more about the goal of the session, share ideas, and ask questions. We believe this will have a positive impact on student participation.

## Conclusions

Our study provides some evidence that case-based training of medical students beginning as early as the first year of the curriculum regarding issues that affect the health of LGBT seniors, can improve attitudes, and sensitize them to the unique needs of this population. Following the session, the students reached out to the research team and facilitators to request more educational resources about the topics covered and to receive further training in this field of study. Our study data demonstrate the effectiveness of the small-group, case-based discussion approach involving members of the LGBT community as facilitators to enhance the cultural competency of the medical students. Our study also highlights that early integration of the LGBT curriculum in medical school curricula has the potential to reduce the incidence of adverse healthcare interactions for these patients, especially seniors who are among the most vulnerable groups. Future work with these students will include additional senior LGBT proficiency training to be offered longitudinally across the curriculum. We hope to utilize the same pool of facilitators to continuously implement and integrate LGBT health curricula in diverse academic settings within our medical school.

## Appendices

APPENDIX A: Pre and Post session Surveys

Pre-session Survey

Please answer the following demographic questions:

1.     Enter your name: ________________

2.     Please indicate which gender you identify with. 

a.      Male

b.     Female 

c.      Transgender 

d.     Other

e.      Prefer not to answer

3.      Please indicate your sexual orientation.  

​​​​a.      Heterosexual

b.     Gay 

c.      Lesbian

d.     Bisexual

e.     Queer

f.      Other

g.     Prefer not to answer 

4.      Please select your age range: 

a.      24 or below

b.     25 - 29 

c.      30 - 34 

d.     35 - 44

e.      45 - 54

f.      55 - 64

g.     65 and over

h.     Prefer not to answer 

5.      Which ethnicity/race describes you?

a.      Hispanic

b.     Black, non-Hispanic

c.      American Indian/Alaskan Native

d.     White, non-Hispanic

e.      Asian/Pacific Islander

f.      Other

g.     Prefer not to answer 

6.      Please indicate the extent to which you agree or disagree that each of the following factors is a strong determinant of the health status of an individual or population (Table 3).

**Table 3:** Form for indicating factors/determinants of the health status of an individual or population

	Strongly Agree 5	Agree 4	Undecided 3	Disagree 2	Strongly Disagree 1
Lifestyle					
Access to health care					
Independent/Assisted Living (Housing)					
Employment					
Educational Status					
Ageism					
Sexism					
Racism					
Classism					
Ableism					
Heterosexism					
Religion					

7.      Please indicate the extent to which you agree or disagree with each of the following statements:

**Table 4:** Form to indicate agreement/disagreement with certain statements

	Strongly Agree 5	Agree 4	Undecided 3	Disagree 2	Strongly Disagree 1
I am confident in my knowledge about the systemic barriers to health faced by LGBTQ+ individuals					
I am confident in my knowledge about the unique health issues and disparities for LGBTQ+ individuals					
I am confident in my knowledge about good practices for promoting culturally competent care for LGBTQ+ individuals					
I am confident in my knowledge about common inappropriate practices that prevent culturally competent care for LGBTQ+ individuals					

Post-session Survey

Participation in this survey is completely voluntary and in no way affects your grades. The goal of this survey is to assess students’ attitudes and confidence regarding LGBTQ+ healthcare and the activity conducted today. Please contact a member of the research team for any questions or concerns. This survey will be used to aid in curriculum development at NSU MD and for publication.

Please answer the following demographic questions:

1.  Enter your name: ________________

2.     Please indicate which gender you identify with. 

a.      Male

b.     Female 

c.      Transgender 

d.     Other

e.      Prefer not to answer

3.      Please indicate your sexual orientation.  

​​​​a.      Heterosexual

b.     Gay 

c.      Lesbian

d.     Bisexual

e.     Queer

f.      Other

g.     Prefer not to answer 

4.      Please select your age range: 

a.      24 or below

b.     25 - 29 

c.      30 - 34 

d.     35 - 44

e.      45 - 54

f.      55 - 64

g.     65 and over

h.     Prefer not to answer 

5.      Which ethnicity/race describes you?

a.      Hispanic

b.     Black, non-Hispanic

c.      American Indian/Alaskan Native

d.     White, non-Hispanic

e.      Asian/Pacific Islander

f.      Other

g.     Prefer not to answer 

6.     Please indicate the extent to which you agree or disagree that each of the following factors is a strong determinant of the health status of an individual or population:

**Table 5:** Form for indicating factors/determinants of the health status of an individual or population

	Strongly Agree 5	Agree 4	Undecided 3	Disagree 2	Strongly Disagree 1
Lifestyle					
Access to health care					
Independent/ Assisted Living (Housing)					
Employment					
Educational Status					
Ageism					
Sexism					
Racism					
Classism					
Ableism					
Heterosexism					
Religion					

7.     Please indicate the extent to which you agree or disagree with each of the following statements:

**Table 6:** Form to indicate agreement/disagreement with certain statements

	Strongly Agree 5	Agree 4	Undecided 3	Disagree 2	Strongly Disagree 1
I am confident in my knowledge about the systemic barriers to health faced by LGBTQ+ individuals					
I am confident in my knowledge about the unique health issues and disparities for LGBTQ+ individuals					
I am confident in my knowledge about good practices for promoting culturally competent care for LGBTQ+ individuals					
I am confident in my knowledge about common inappropriate practices that prevent culturally competent care for LGBTQ+ individuals					

8.     Please write any questions you may have regarding the provision of culturally competent and affirming care for LGBTQ+ individuals?

__________________________________________________________________

9.     Please provide any feedback about how this activity may have contributed to your understanding of the healthcare issues faced by LGBTQ+ individuals?

__________________________________________________________________

Some survey items were adopted with modifications from Gazvy et al 2019: Gavzy, S. J., Berenson, M. G., Decker, J., Domogauer, J., Alexander, A., Pulaski, M., Soto-Greene, M., Sánchez, N., & Sánchez, J. P. (2019). The Case of Ty Jackson: An Interactive Module on LGBT Health Employing Introspective Techniques and Video-Based Case Discussion. MedEdPORTAL: The journal of teaching and learning resources, 15, 10828. https://doi.org/10.15766/mep_2374-8265.10828

APPENDIX B: Assessment Rubric

**Table 7:** Assessment Rubric

	Professionalism		Interpersonal Skills and Communication		Practice Based Learning and Improvement		General Comments
Student Name	Respectful of peers, faculty, and LGBTQ guests (Please identify the specific group such as “peers” or “faculty” in your comments)		1) Appropriateness of engagement with the LGBTQ guests and team members (Openness to ideas presented by others, balanced active listening, clear verbal and non-verbal communication, conflict management) 2) Appropriateness of participation (Quiet, domineering, disrespectful, balanced)		1) Quality of contribution to prompt questions and drives discussions 2) Response to feedback or self-evaluation (if applicable)		Any other concerns or accolades
	1– 5 rating	Comments	1– 5 rating	Comments	1– 5 rating	Comments	
